# Taurine ameliorates sensorimotor function by inhibiting apoptosis and activating A2 astrocytes in mice after subarachnoid hemorrhage

**DOI:** 10.1007/s00726-024-03387-5

**Published:** 2024-04-14

**Authors:** Chunlei Yang, Zhiwen Jiang, Xinjie Gao, Heng Yang, Jiabin Su, Ruiyuan Weng, Wei Ni, Yuxiang Gu

**Affiliations:** 1 Department of Neurosurgery, Huashan Hospital, Fudan University, Shanghai, 200040 China; 2 National Center for Neurological Disorders, Shanghai, 201107 China; 3 Neurosurgical Institute of Fudan University, Shanghai, 201107 China; 4 Shanghai Key Laboratory of Brain Function and Restoration and Neural Regeneration, Shanghai, 200052 China; 5 Institute of Science and Technology for Brain-Inspired Intelligence, Fudan University, Shanghai, 200433 China

**Keywords:** Subarachnoid hemorrhage, Neuroprotection, Taurine, Astrocytes

## Abstract

Subarachnoid hemorrhage (SAH) is a form of severe acute stroke with very high mortality and disability rates. Early brain injury (EBI) and delayed cerebral ischemia (DCI) contribute to the poor prognosis of patients with SAH. Currently, some researchers have started to focus on changes in amino acid metabolism that occur in brain tissues after SAH. Taurine is a sulfur-containing amino acid that is semi-essential in animals, and it plays important roles in various processes, such as neurodevelopment, osmotic pressure regulation, and membrane stabilization. In acute stroke, such as cerebral hemorrhage, taurine plays a neuroprotective role. However, the role of taurine after subarachnoid hemorrhage has rarely been reported. In the present study, we established a mouse model of SAH. We found that taurine administration effectively improved the sensorimotor function of these mice. In addition, taurine treatment alleviated sensorimotor neuron damage and reduced the proportion of apoptotic cells. Furthermore, taurine treatment enhanced the polarization of astrocytes toward the neuroprotective phenotype while inhibiting their polarization toward the neurotoxic phenotype. This study is the first to reveal the relationship between taurine and astrocyte polarization and may provide a new strategy for SAH research and clinical treatment.

## Introduction

Subarachnoid hemorrhage (SAH) is a severe acute cerebrovascular disease that is associated with very high mortality and disability rates (Claassen and Park 2022). There are multiple causes of SAH; the most prominent cause of SAH is the rupture of intracranial aneurysms, which accounts for approximately 85% of the nontraumatic SAH cases (Macdonald and Schweizer 2017). Other causes of SAH include intracranial vascular arteriovenous malformations, cranial trauma, atherosclerosis, and moyamoya disease. A survey by the World Health Organization showed that the incidence of SAH is highest in Japan and Finland, at approximately 20/100,000 person-years (Nieuwkamp et al. 2009). The incidence of SAH in China is lower, approximately 2/100,000 person-years, which may be explained by the low proportion of postmortem autopsies that are performed on Chinese patients; thus, the true incidence of SAH may be underestimated. Even if a patient survives the first bleeding, secondary bleeding and very high disability rates severely affect patient prognosis (Stienen et al. 2018). The neurological status of a patient after onset, especially the patient’s level of consciousness, is the most important factor that determines the prognosis of SAH. Therefore, protection of neurological function after SAH is the focus of SAH research.

To date, research on SAH has focused on early brain injury (EBI) (Lauzier et al. 2023) and delayed cerebral ischemia (DCI) (Geraghty and Testai 2017). EBI is defined as direct damage to the brain within 72 h after SAH and various subsequent pathophysiological changes (Zeyu et al. 2021). Many pathophysiological processes are associated with EBI, including blood‒brain barrier disruption, increased intracranial pressure, cerebral ischemia, impaired microcirculation, brain edema, oxidative stress, neurovascular inflammatory responses, and neuronal apoptosis and autophagy (Cahill et al. 2006; Fujii et al. 2013; Wu et al. 2022; Zhang et al. 2022). These pathophysiological processes occur immediately after the onset of SAH and rapidly worsen within a short period of time. Delayed cerebral ischemia occurs within 4–10 days after SAH, resulting in hypoperfusion of brain tissue, which leads to a poor prognosis (Veldeman et al. 2016; Dodd et al. 2021). The current approach for the clinical management of SAH patients strongly focuses on delayed cerebral ischemia after SAH (Balança et al. 2022). The current dilemma in the management of subarachnoid hemorrhage has led us to rethink whether other factors are involved in the pathophysiology of SAH that have not yet been considered but that contribute to poor prognosis. The essence of subarachnoid hemorrhage is the rapid accumulation of blood in the subarachnoid space due to various factors, and blood, as an exogenous stimulus, triggers a series of subsequent pathophysiological processes. This initial pathogenic factor leads to secondary brain injury and the associated metabolic changes after SAH (Chen et al. 2022). Therefore, metabolomic alterations after SAH may elucidate the pathophysiological processes that occur after SAH, and studies targeting metabolites after SAH may provide new approaches for treating secondary brain injury after SAH (Lasica et al. 2022).

Taurine is a sulfur-containing amino acid that is semi-essential in animals, and it can be produced by the liver and kidneys or ingested from outside the body (HuxTable 1992). Taurine is highly enriched in tissues and organs, such as the retina, brain tissue, heart, and placenta (Yang et al. 2022). Taurine plays important roles in various processes, such as neurodevelopment, osmotic pressure regulation, membrane stabilization, reproductive system stabilization, myocardial regulation and inflammation regulation (Silva et al. 2021). During acute stroke, taurine may also play a neuroprotective role (Zhao et al. 2018). Taurine exerts protective effects on different neurological diseases through multiple pathways. However, the role of taurine after subarachnoid hemorrhage has rarely been reported.

## Materials and methods

### SAH mouse model

C57BL/6 mice (8–10 weeks old, approximately 25 g) were obtained from the Laboratory Animal Center of Fudan University. All the animal experiments were randomized and performed in a double-blinded manner. The SAH model was established through endovascular puncture. In brief, the mice were anesthetized via inhalation of isoflurane. The mice were subjected to a cervical midline incision, and a sharp 6-0 nylon suture was inserted into the right internal carotid artery through the bifurcation of the external carotid artery and common carotid artery. The suture was advanced forward until resistance was felt at the bifurcation of the anterior and middle cerebral arteries. The suture was then advanced further to puncture the vessel and then immediately removed. Deep breathing was observed in the mice. In the sham operation, the suture was inserted into the right carotid artery, but no blood vessels were punctured. After the suture was removed, the skin incision was sutured. All the experiments were performed following ethical norms and animal welfare principles and were approved by the Animal Ethics Committee of Fudan University.

### Experimental design and drug administration

106 mice were used in this study. All mice were randomly divided into four groups: the Sham group, the Sham + taurine group, the SAH group, the SAH + taurine group. According to the literature, taurine was dissolved in saline and intraperitoneally injected into the mice at a concentration of 100 mg/kg immediately after SAH was established (Silva et al. 2021). Taurine powder was purchased from Sigma Corporation.

### Modified Garcia score

The modified Garcia score was calculated at 1, 3, and 7 days after the SAH model was established. The scoring was double-blinded, with the scorer and statistician being nonresearcher modelers, and subjective factors were excluded as much as possible. The mice were scored in six main areas: spontaneous activity in the cage, left–right symmetry of limb movement, left–right symmetry of tail movement, climbing ability, response to touch on one side of the body, and response to touch of the whiskers. Each item was assigned a score of 0–3, for a total possible score of 18.

### Foot fault test

A 40 cm long, 20 cm wide and 50 cm high stainless-steel frame was prepared. The top surface was a square grid that was woven with a 3-mm diameter wire, the grid was a square with sides that were 2 cm in length, and the middle of the grid was hollow. A camera was placed approximately 40 cm from the stainless-steel frame so that the lens was aligned and included the upper wire mesh. The mice were placed on the wire mesh for 3 min each day for three days prior to SAH model establishment to allow them to acclimate to the experimental conditions. The mice were placed on the wire mesh for 3 min each (or at least 100 steps) at 1, 3, and 7 days after SAH model establishment. The mouse’s gait was recorded with a video camera, and the ratio of the number of empty steps to the total number of steps was subsequently calculated; this value was considered the misstep rate.

### Rotarod test

The mice were placed on a rotarod 3 times a day for 3 days prior to SAH model establishment to allow them to become familiar with and adapt to the rotarod in advance. The starting speed was set to 5 r/min for 300 s, and the speed was gradually increased to a final speed of 40 r/min. The mice were tested at 1, 3, and 7 days after SAH model establishment, three times a day, and the durations for which the mice remained on the rotarod were recorded and averaged. The device was cleaned with an alcohol spray bottle after each mouse to avoid the effect of odor on mouse behavior.

### Western blotting

Brain tissues were harvested from the sensorimotor cortex of the mice 3 days after SAH. The tissues were lysed with RIPA assay buffer, and 20 µg of proteins were separated via 4–20% SDS–PAGE and transferred to polyvinylidene difluoride membranes. After blocking, the membranes were incubated overnight with the indicated primary antibodies at 4 °C. The following primary antibodies were used: anti-β-actin (1:10,000; Proteintech), anti-TauT (1:1000; Abcam), anti-bax (1:1000; Proteintech), anti-bcl-2 (1:1000; Proteintech), and anti-COX IV (1:1000; Proteintech) antibodies. Chemical reactions were detected with an ECL system. The scanned images were analyzed with ImageJ software.

### Immunofluorescence staining

Brain samples were fixed with 4% paraformaldehyde. Then, brain sections (5 µm) of the cortex were incubated with the following primary antibodies at 4 °C overnight: anti-NeuN (1:500; Abcam), anti-TauT (1:100; Abcam), anti-cleaved caspase 3 (1:500; CST), anti-S100A10 (1:500; Proteintech), anti-S100B (1:500; CST) antibodies. The samples were then incubated with Alexa Fluor 488/594-conjugated secondary antibodies (1:500) at 37 °C for 2 h. 4′,6-Diamidino-2-phenylindole staining was then used to label the cellular nuclei. Finally, images of the sensorimotor cortex were captured by fluorescence microscopy.

### Nissl staining

Mouse brain tissue sections were washed and then immersed in 0.5% cresyl violet solution (Sigma‒Aldrich). The sections were then sequentially dehydrated and cleared in xylene for 3 min. The brain tissue sections were mounted with Permount and coverslipped. Random fields of the temporal cortex and the hippocampus were observed under a light microscope, and the number of surviving neurons was counted by an investigator who was blinded to the treatment group. All the images were taken from the sensorimotor cortex.

### Hematoxylin and eosin (H&E) staining

H&E staining was used for histomorphological analysis. Briefly, brain slices were placed in hematoxylin and eosin solution, redehydrated in gradient ethanol solutions, treated with dimethylbenzene, and covered with coverslips. The pathological images were scanned with a digital pathological section scanner. All the images were taken from the sensorimotor cortex.

### Terminal-deoxynucleotidyl transferase dUTP nick-end labeling (TUNEL) staining

Cell apoptosis was detected using TUNEL staining according to the manufacturer’s protocol (Share Bio). Next, slides were counter-stained with 40,6-diamidino-2-phenylindole (DAPI). Three microscope fields of TUNEL-positive cells in sensorimotor cortex were chosen and imaged.

### Statistical analysis

GraphPad Prism 8 was used to analyze the data. All the data are presented as the mean ± standard deviation. Immunofluorescence of the western blotting images was analyzed by one-way analysis of variance followed by Tukey’s post hoc analysis. The neurological scores and ethology were analyzed using a Kruskal–Wallis one-way analysis of variance on ranks followed by Dunn’s post hoc test. *p* < 0.05 was considered to indicate a significant difference.

## Results

### Taurine transporter expression increases in neurons and astrocytes after SAH

**Fig. 1:**
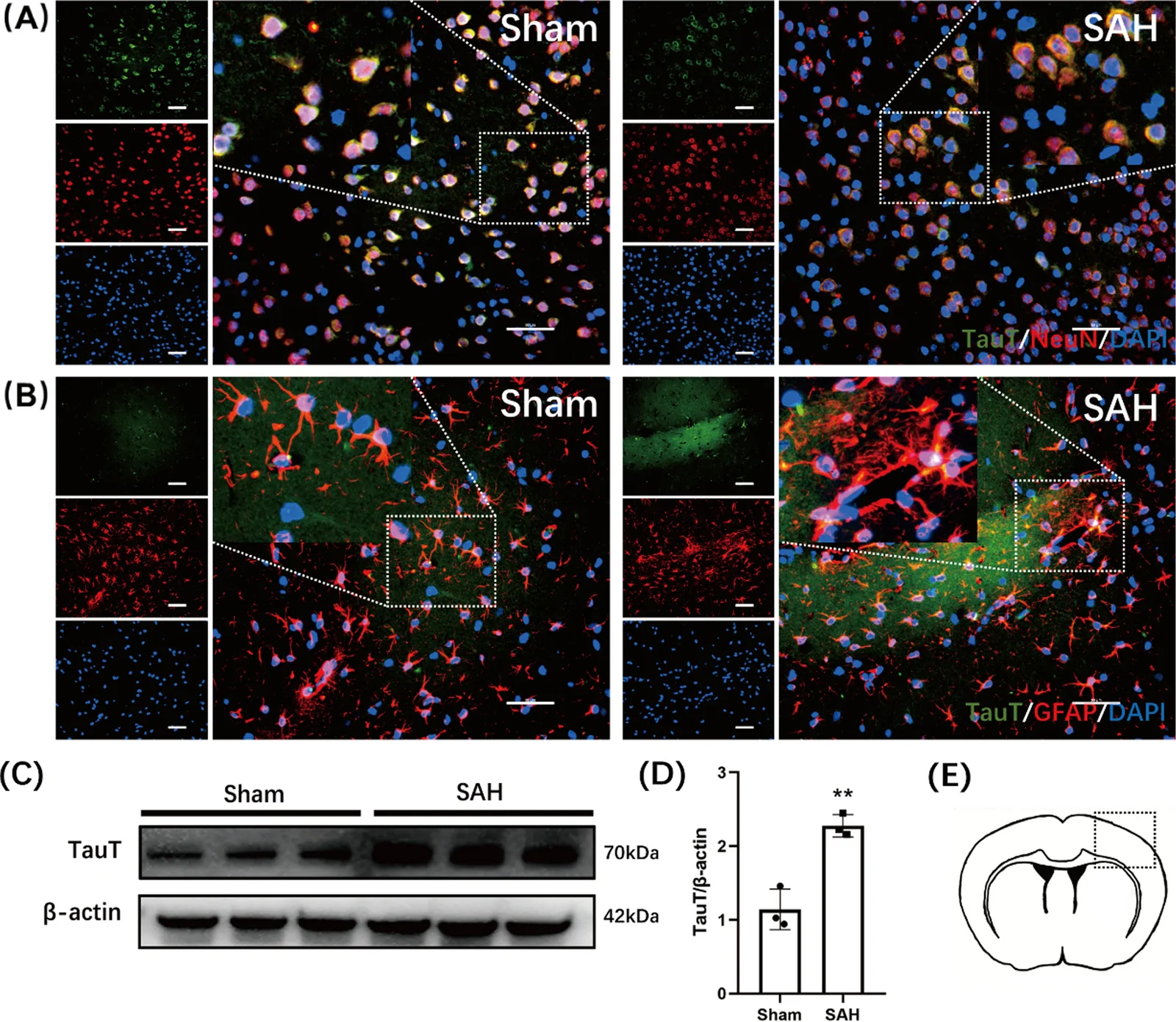
Expression of TauT in the mice cortex. **A** Expression of TauT in the cortex of mice. TauT was co-labeled with NeuN which represent TauT is expressed in neurons. *Scale bar*: 50 μm. **B** Expression of TauT in astrocytes. TauT was also co-labeled with GFAP. **C** Western blot shows the expression of TauT was significantly higher in the SAH group compared to the Sham group. **D** Statistical graph of TauT protein expression in two groups of mice. ** represents *p* < 0.01. **E** The location of the slices

In the central nervous system, neurons are not able to synthesize taurine to meet metabolic needs. Instead, taurine is taken up by neurons mainly via a specific transporter named the taurine transporter (TauT). TauT was found to play crucial roles in the blood-testicular barrier and retina. However, few studies have focused on TauT in the CNS. In our present study, TauT labeling colocalized with that of NeuN and GFAP, which indicated that TauT was expressed in neurons and astrocytes (Fig. 1a, b). The position of the slices is shown in Fig. 1e. To investigate whether the expression level of TauT changes after SAH, we used Western blotting to measure TauT protein expression. The results showed that after SAH, TauT expression was increased compared with that in the sham group, indicating compensatory overexpression. These results indicate that TauT is expressed on the membrane of neurons. In addition, after SAH, the expression of TauT is increased (Fig. 1c, d).

### Taurine ameliorates neurological scores and neurobehavior after SAH

**Fig. 2:**
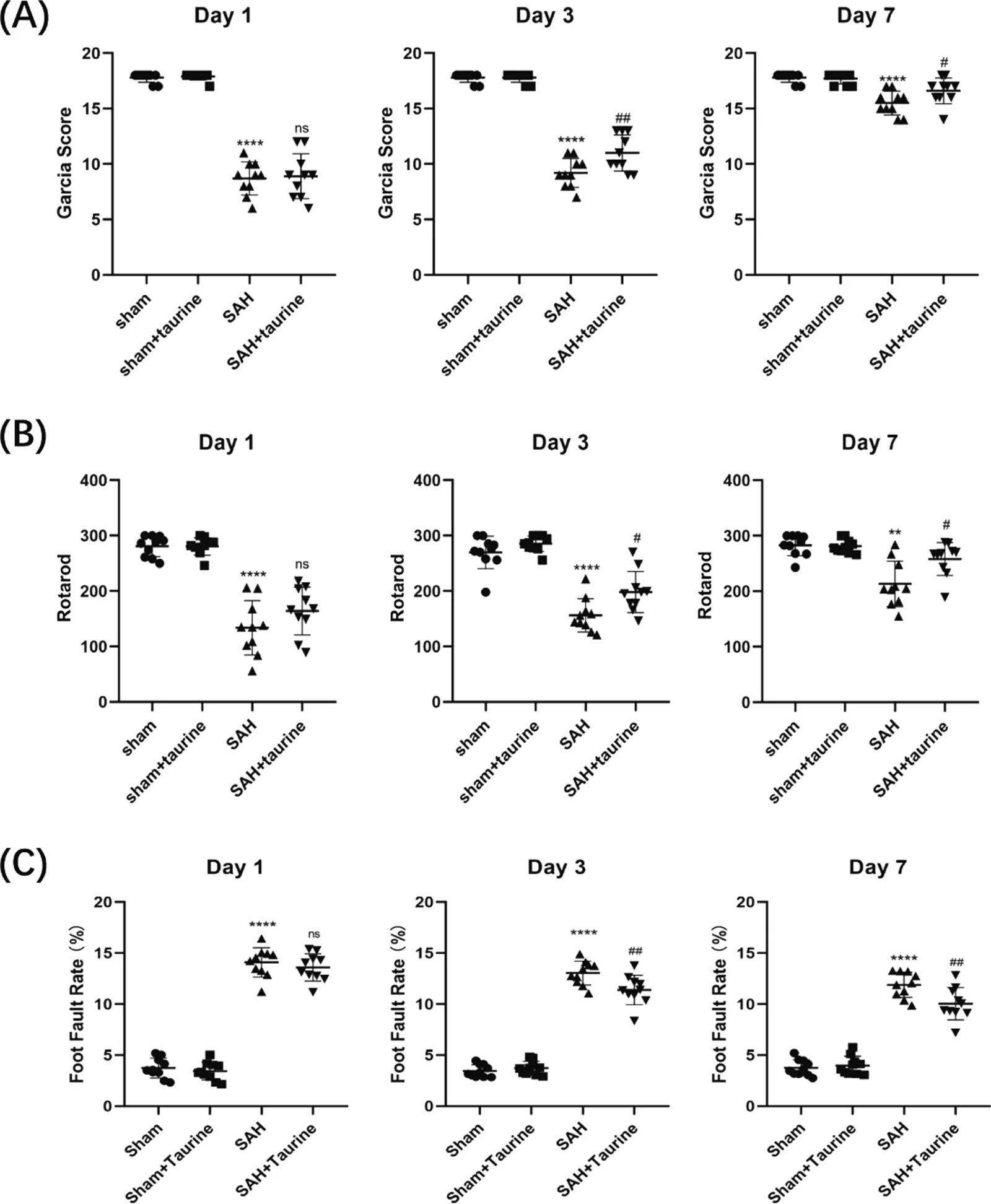
Taurine improves neurobehavioral scores and sensorimotor functions after SAH in mice. **A** Modified Garcia scores at different time points after SAH establishment in mice. At 1 day after SAH, the SAH group had significantly lower scores than the control group, but the scores did not improve after 1 day of taurine treatment. There was a significant increase in the Garcia score after 3 days of taurine treatment. Seven days after SAH, the mice in the SAH + taurine group also had significantly lower scores than did those in the SAH group. **B** Rotarod test with SAH model mice. At 1 day, the mice in the SAH group had a significantly shorter duration on the rotarod, with a mean value of approximately 120 s. The addition of taurine at 1 day did not prolong the time spent on the rotarod. At 3 days after SAH, the duration spent by the taurine-treated mice on the rotarod was significantly longer than that of the SAH group. Seven days after SAH, the duration spent by the mice on the rotarod was significantly increased, and the increase in the time was more obvious in the taurine-treated group. **C** Foot fault test of SAH model mice. There was no significant decrease in the misstep rate in the Sham or Sham + taurine groups after SAH. At 1 day, the mice in the SAH group had a significantly higher percentage of missteps, reaching approximately 15%. However, at 3 and 7 days, the misstep rate of the mice in the taurine-treated group was significantly lower than that in the SAH group, indicating that taurine improved motor function in the acute phase after SAH in mice to a certain degree. * represents SAH vs. Sham, # represents SAH vs. SAH + taurine. **** represents *p* < 0.0001, ** represents *p* < 0.01, ^##^ represents *p* < 0.01, and ^#^ represents *p* < 0.05

To evaluate the effect of taurine after SAH, we used a modified Garcia score to assess the degree of injury in the mice. Our data showed that on 1, 3, and 7 days after SAH was established in the model group, the Garcia score was significantly lower than that in the sham group. One day after taurine administration, the Garcia score did not improve. On 3 and 7 days after SAH, the neurological score was significantly improved (Fig. 2a). Next, we examined sensorimotor function by determining the foot fault rate and analyzing the rotarod test results. One day after SAH, motor function was distinctly decreased, but taurine did not play a protective role. Three days after SAH, the survival time of the SAH + taurine group was obviously prolonged compared to that of the SAH group (Fig. 2b). The foot fault rates exhibited similar trends. One day after SAH, the SAH group and the SAH + taurine group had the highest foot fault rates. Three days after SAH, taurine significantly decreased the number of foot faults (Fig. 2c). These results indicate that taurine ameliorates the neurological score and neurobehavior of mice 3 days after SAH.

### Taurine mitigates injury to the sensorimotor cortex after SAH

**Fig. 3:**
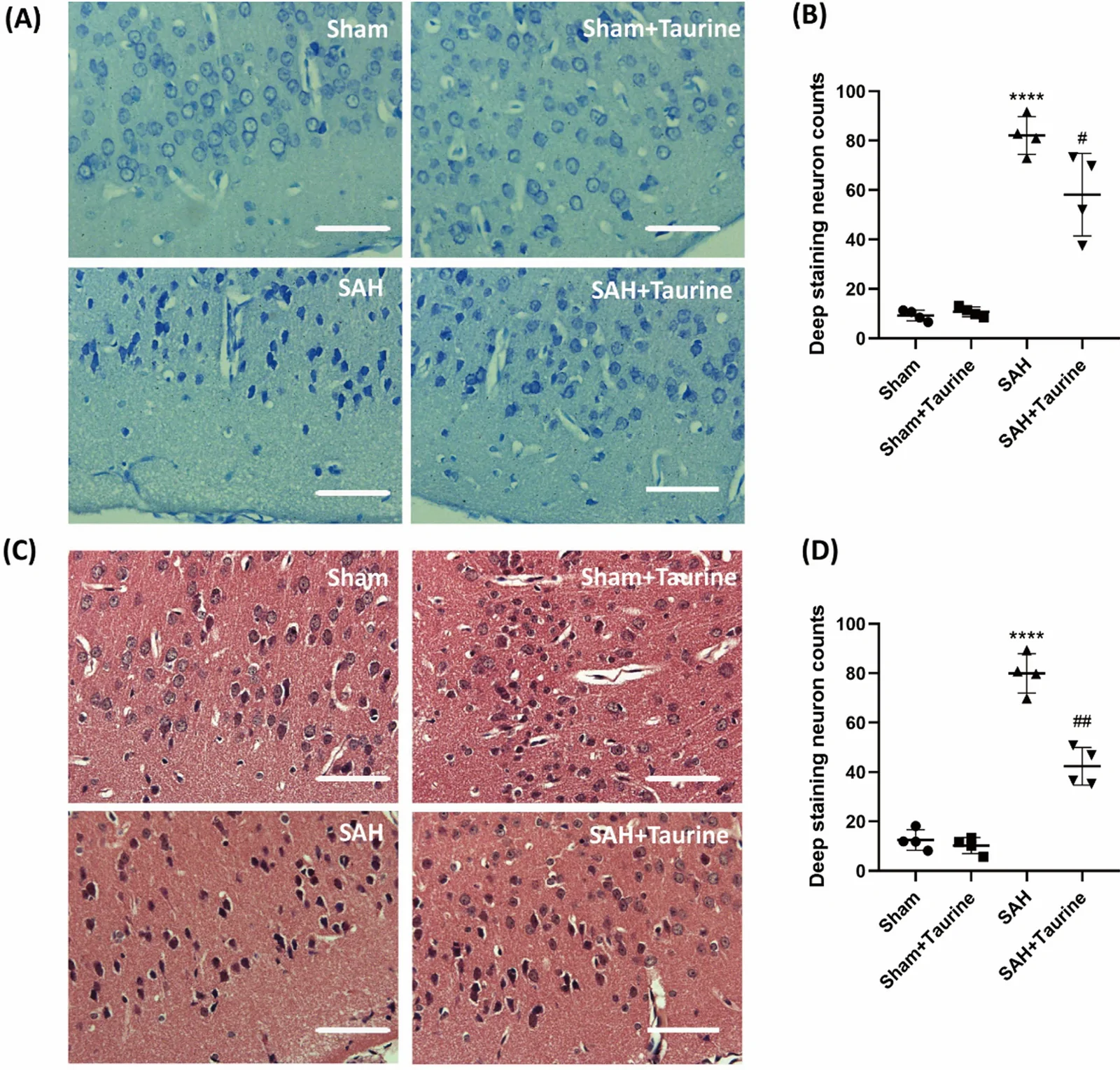
H&E staining and Nissl staining of the mouse cortex. **A** and **B** The number of cortical cells was higher in the Sham and Sham + taurine groups, the morphology was normal, and deep staining of normal Nissl bodies in the cytoplasm was observed. The cells were irregular and wrinkled. The morphology of cortical cells was restored in the SAH + taurine group. **C** and **D** In the Sham group and Sham + taurine group, the cortical structure was clear, and the number and morphology of the cells were normal. In the SAH group, the number of deep staining cells was significantly increased, and the cells were irregular in morphology. The number and morphology of cells in the sensorimotor cortex of mice were restored after the addition of taurine. *Scale bar*: 25 μm

To examine the neuroprotective effect of taurine on the structure of the sensorimotor cortex, we used Nissl staining and H&E staining. Nissl staining revealed that the structure of the cortex was severely damaged after SAH, and the number of deep staining neurons was significantly increased (Fig. 3a, b). In addition, H&E staining was used to observe the morphology of single neurons and cortex structure. We found that the number of deep staining neurons was increased after SAH, while the administration of taurine reduced the number of deep staining neurons (Fig. 3c, d). In summary, taurine reduced the number of deep staining neurons and ameliorates the structure of the sensorimotor cortex.

### Taurine alleviate apoptosis in the sensorimotor cortex

**Fig. 4:**
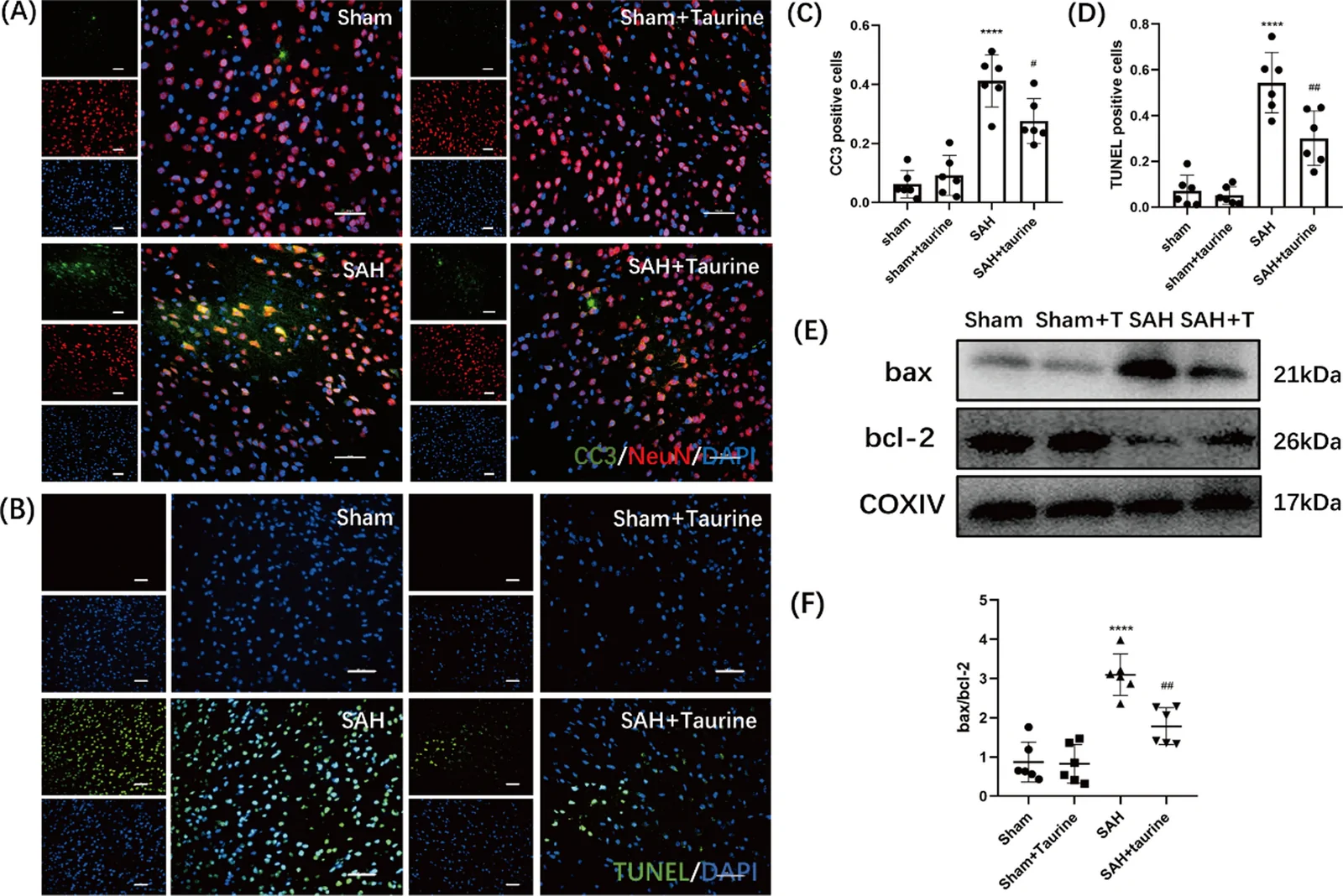
Detection of apoptosis in the mouse cortex. **A** Immunofluorescence staining of paraffin sections of the mouse cortex from the four groups. There were very few cleaved caspase 3-positive cells in the Sham and Sham + taurine groups, and a large number of cleaved caspase 3-positive neurons were observed in the SAH group. In the taurine-treated group, the proportion of CC3-positive cells was decreased. *Scale bar*: 50 μm. **B** TUNEL staining of cortices from the four groups of mice. After SAH, the number of TUNEL-positive cells was significantly increased. After the addition of taurine, the number of TUNEL-positive cells was decreased. **C** Statistical analysis of cleaved caspase 3 staining. **D** Statistical analysis of the proportion of TUNEL-positive cells. **E** Western blotting analysis of mouse cortical protein expression. In the SAH group, the protein expression of bax and cleaved caspase 3 was increased, while that of bcl-2 was decreased. After taurine injection, bax and cleaved caspase 3 expression was decreased, and bcl-2 expression was increased compared with that in the SAH group. **F** Statistical analysis of the bax/bcl-2 ratio in the cortex of the four groups of mice. **G** Statistical analysis of the cortical cleaved caspase 3/β-actin ratio in the four groups of mice. * represents SAH vs. Sham, ^#^ represents SAH + taurine vs. SAH, ****p < 0.0001; ^#^*p* < 0.05; ^##^*p* < 0.01

Taurine has been reported to play crucial roles in many diseases. However, whether taurine can reduce apoptosis in the sensorimotor cortex after SAH is still unknown. Here, we measured the expression of cleaved caspase 3, which is a marker of apoptosis. After SAH, the expression of cleaved caspase 3 was visibly increased. Taurine supplementation reduced the number of apoptotic neurons compared with that in the SAH group (Fig. 4a, c). Terminal-deoxynucleotidyl transferase-mediated nick-end labeling (TUNEL) is a sensitive marker of apoptosis and reveals DNA breaks in the nucleus. Our data showed that the percentage of TUNEL-positive cells was obviously increased in the SAH group. Moreover, taurine significantly decreased the percentage of apoptotic cells (Fig. 4b, d). Then, we measured the expression of several proteins that are related to apoptosis. After SAH, the expression of bax was increased, while the expression of bcl-2 was decreased. Taurine administration reduced bax expression and increased bcl-2 expression (Fig. 4e, f). Overall, taurine ameliorates apoptosis in the sensorimotor cortex to protect CNS cells from injury.

### Taurine facilitates the polarization of reactive astrocytes toward the neuroprotective phenotype

**Fig. 5:**
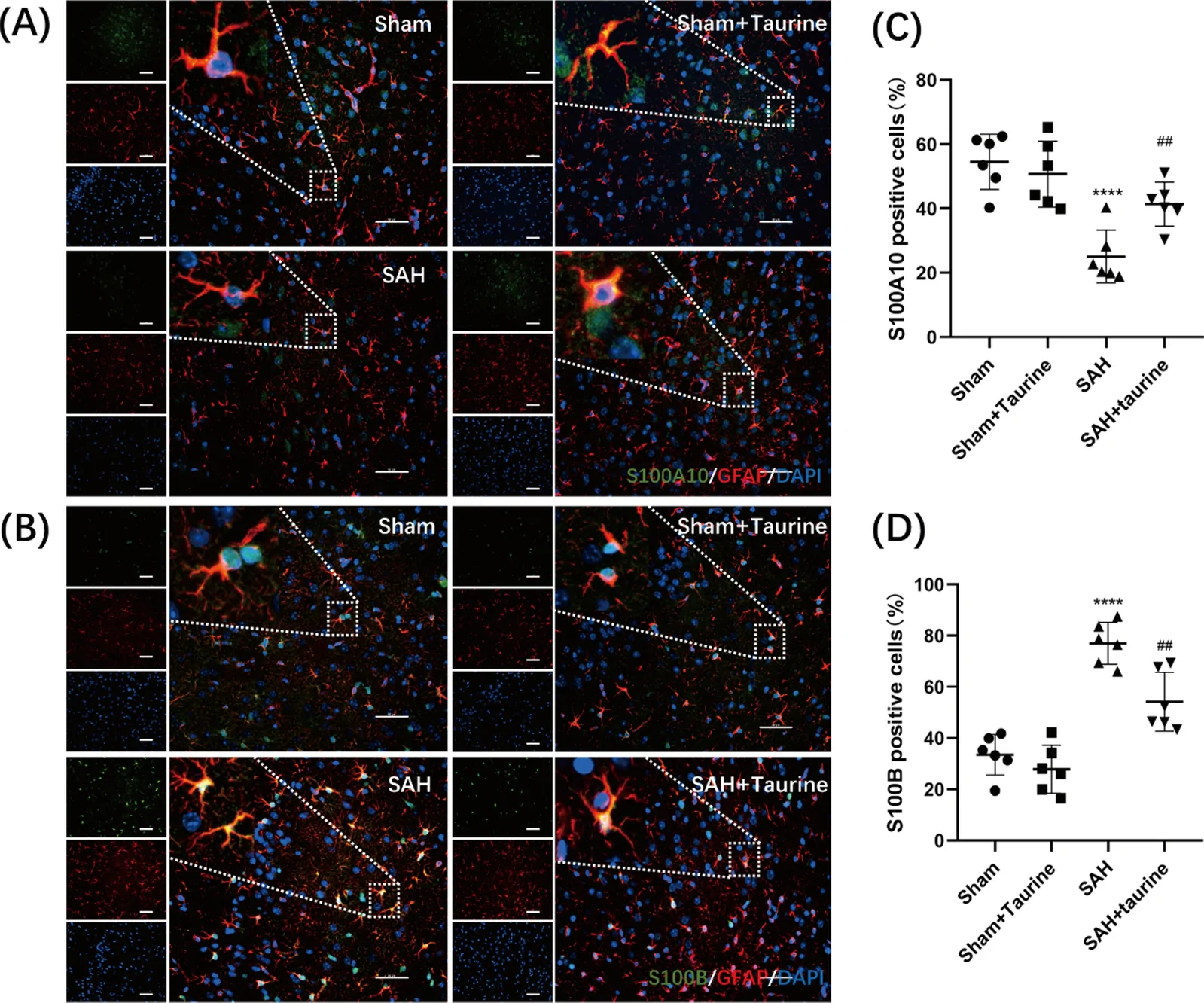
Polarization of astrocytes in mouse cortical tissue. **A** The number of cortical astrocytes in the Sham and Sham + taurine groups was lower, with small cytosolic volume and few branches, indicating an inactivated state. After SAH, the proportion of astrocytes that expressed with S100A10 and GFAP was significantly reduced. The proportion of S100A10-positive astrocytes was increased again after the addition of taurine. *Scale bar*: 50 μm. **B** The proportion of S100B-positive astrocytes in the Sham group was lower. The proportion of S100B-positive astrocytes decreased after taurine supplementation. *Scale bar*: 50 μm. **C** Proportion of S100A10-positive astrocytes relative to total astrocytes. **D** Proportion of S100B-positive astrocytes relative to total astrocytes. * represents SAH vs. Sham, ^#^ represents SAH + taurine vs. SAH. **** represents *p* < 0.0001, ^##^ represents *p* < 0.01

After acute brain injury or central nervous system degeneration, astrocytes quickly respond by undergoing a stereotypical pattern of molecular and morphological alterations; these cells are called reactive astrocytes. There are two main types of reactive astrocytes, A1 astrocytes and A2 astrocytes. A1 astrocytes are harmful to neurons, while A2 astrocytes secrete neurotrophic factors to protect neurons. Our data showed that after SAH, astrocytes became active because the level of GFAP increased. In addition, the proportion of A1 astrocytes was significantly increased. After taurine supplementation, the expression of S100A10 was increased, while the level of S100B was decreased, indicating that the percentage of A1 astrocytes decreased while the percentage of A2 astrocytes increased (Fig. 5).

## Discussion

Subarachnoid hemorrhage (SAH), especially aneurysmal subarachnoid hemorrhage, is a serious acute cerebrovascular disease that leads to high mortality rates (Neifert et al. 2021). The incidence of SAH has decreased worldwide in recent decades, probably due to a better understanding of the disease and improved lifestyle habits, such as the management of hypertension and the restriction of smoking and alcohol (Long et al. 2017). Nevertheless, the rates of early morbidity and mortality remain high among SAH patients, and it has been reported that up to a quarter of patients with aneurysmal SAH die without reaching the hospital or die in the emergency room. Even when patients are fortunate enough to survive the first bleeding event, the incidence of subsequent secondary bleeding is also high (Claassen and Park 2022). In addition, patients with SAH have a high rate of disability, which severely affects their quality of life and that of their families (Steiner et al. 2013). Therefore, reducing neurological damage and improving the long-term prognosis of SAH patients have been the focus of SAH treatment.

In previous years, the mainstream view was that delayed cerebral ischemia (DCI) after SAH was the main cause of the poor prognosis of SAH patients (Rass and Helbok 2021). DCI manifests as delayed neurodegeneration and focal deficits that can further develop into cerebral infarction (Suzuki et al. 2021). Investigators have suggested that DCI is associated with the development of cerebral vasospasm after SAH and is a direct result of arterial stenosis (Li et al. 2019). However, subsequent studies have shown that controlling cerebral vasospasm after SAH onset is not effective in ameliorating DCI or improving the prognosis of SAH patients (Topkoru et al. 2017). Recent studies have, therefore, begun to distinguish between these two events and suggest that DCI may be the result of several underlying and interrelated mechanisms. Early brain injury (EBI) after SAH is receiving increased attention from scientists due to the large amount of blood that enters the subarachnoid space very soon immediately after SAH onset, resulting in sustained elevated intracranial pressure, disruption of the blood‒brain barrier, and continued irritation of the meninges by blood as a foreign body (Cahill and Zhang 2009). EBI usually occurs within 72 h after SAH. Along with the disruption of the blood‒brain barrier, neurons in brain tissue also undergo a series of pathophysiological changes, including neuroinflammation, apoptosis and autophagy, endothelial damage, and excitotoxic effects of blood and its breakdown products on neurons (Lucke-Wold et al. 2016; Yang et al. 2017; Zhao et al. 2022). These effects coexist and interact with each other to further exacerbate brain injury after SAH. Therefore, the prevention and treatment of EBI and DCI can improve the prognosis of SAH patients to some extent. Currently, researchers have focused on metabolic changes in the CSF after SAH, and they found that the levels of various amino acids, including taurine, in CSF increase after SAH.

Taurine is a sulfur-containing β-amino acid. Although taurine is not directly involved in protein composition, it plays several roles in normal physiological activities (HuxTable 1992). Taurine is considered a cytoprotective molecule because of its ability to maintain a normal electron transport chain, regulate autophagy, enhance antioxidant responses, increase membrane stability, eliminate inflammation, and prevent calcium accumulation (Surai et al. 2021; Duan et al. 2023; Qu et al. 2023). Due to its unique sulfonic acid composition, taurine also regulates cellular osmotic pressure, modulates ion movement and binds to bile acids to facilitate their circulation (Wang et al. 2022). Some mammals can synthesize taurine endogenously through the liver, but the amount of taurine that is synthesized by these organisms is limited and insufficient to meet the physiological requirements of the body; therefore, additional taurine needs to be acquired through the diet (Menzie et al. 2014). Taurine is considered an important amino acid during development because of the deficiency of endogenous taurine synthesis during the embryonic stage of mammals (Tochitani 2022). Taurine deficiency during the embryonic stage has severe effects on neurodevelopment in infants and children. An experiment that involved taurine supplementation in pregnant mice showed that increasing taurine levels during the perinatal period significantly promoted the learning ability of newborn mice (Gao et al. 2022). In terms of central nervous system disorders, taurine has been shown to have a palliative effect on neurodegeneration. In a streptozotocin-induced model of Alzheimer’s disease, oral administration of taurine protected the rat nervous system from the effects of reduced glutathione levels (Kim et al. 2014). In another study, intraperitoneal administration of taurine prevented increases in the production of lipid peroxidation products. In addition, taurine can restore cognitive function in mouse models of Alzheimer’s disease (AD) by directly binding to the mouse oligomer β-amyloid (Jang et al. 2017). The role of taurine after SAH has been reported in the literature to a lesser extent. Recent studies show that taurine can modulate the GABAB/AKT/GSK3β/β-catenin pathway to attenuate SAH-induced neuronal iron-related death (Liu et al. 2022). In our study, we found that taurine supplementation after SAH significantly improved short-term sensorimotor function in mice. In addition, taurine attenuated neuron damage in the sensorimotor cortex and attenuated neuronal apoptosis. The protective effects of taurine on the sensorimotor cortex could explain the observed improvements in mouse behavior.

Many of the cytoprotective properties of taurine are based on its intracellular levels, which depend on its synthesis and transport, which are controlled mainly by cysteine dioxygenase, cysteine sulfite decarboxylase and the taurine transporter (TauT) (Tappaz 2004). Two types of transporter proteins are involved in the transport of taurine to cells in different tissues: the TauT transporter protein and the PAT1 transporter protein (Baliou et al. 2020). The TauT transporter protein (*SLC6A6*) is considered by researchers to be the main taurine transporter protein and is characterized by ion dependence, high affinity for the substrate and low capacity. Studies have shown that TauT is localized mainly to the cytoplasmic membrane. When the intracellular taurine concentration was reduced using the competitive taurine antagonist β-alanine (Richter et al. 2019), no significant changes in the mitochondrial concentration of taurine were observed, suggesting that taurine transporter proteins are also present on the mitochondrial membrane (Anderson et al. 2009). The use of taurine to treat nuclear swelling and contraction confirmed that the taurine transporter is similarly expressed in the nuclear membrane (Voss et al. 2004). Thus, TauT is localized within cellular membrane structures and can mediate the intake of taurine from the extracellular space into the cell via active transport. As described in the literature, our study showed that in the mouse sensorimotor cortex and hippocampus, TauT is predominantly expressed in cell membranes. When we double-stained cells for TauT and the neuronal marker NeuN, the astrocyte marker GFAP or the microglial marker Iba1 by immunofluorescence, we found that TauT was abundantly colocalized with NeuN and GFAP, but it was not significantly colocalized with Iba1. This demonstrated that the taurine transporter was abundantly expressed in neurons and astrocytes and was expressed at lower levels in microglia. Therefore, the protective effect of taurine treatment on SAH model mice may be achieved through these two types of cells.

Astrocytes are the most abundant cells in the CNS and play integral roles in maintaining normal CNS function (Zhou et al. 2019). In response to CNS injury and disease, astrocytes are activated and become reactive astrocytes, which exhibit different physiological functions (Sofroniew 2020). By genome sequencing and analysis of reactive astrocytes, researchers have shown that astrocytes can be activated into two polarized states, a neurotoxic or proinflammatory phenotype (A1 astrocytes) and a neuroprotective or anti-inflammatory phenotype (A2 astrocytes). Although the simple dichotomy of dividing activated astrocytes into the A1/A2 phenotypes does not reflect the great variety of astrocyte phenotypes, it helps us to understand the responses of astrocytes in various CNS diseases (Escartin et al. 2021). Several researchers have isolated different types of reactive astrocytes from an LPS-induced neuroinflammation model and middle cerebral artery occlusion model, and these researchers purified and genetically analyzed these cells in two categories: A1 and A2 astrocytes (Zamanian et al. 2012). A1 astrocytes lose many normal astrocytic functions and express high levels of many proteins, such as C3, GBP2, and S100B (Changyaleket et al. 2016). A1 astrocytes may also secrete soluble neurotoxins that rapidly kill neurons and mature oligodendrocytes (Miyamoto et al. 2020). Thus, A1 astrocytes may represent a potentially harmful phenotype and exacerbate the severity of disease (Liddelow et al. 2017). In contrast, A2 astrocytes can upregulate neurotrophic or anti-inflammatory genes, promote neuronal survival and growth, and perform a repair function after injury, suggesting that they may play a protective role. A2 astrocytes express high levels of a variety of proteins, such as S100A10, PTX3, and S1Pr3 (Wang and Li 2023). Evidence for the association of the A1/A2 polarization of astrocytes has now been found in models of central nervous system diseases, including white matter injury, ischemic stroke, and spinal cord injury, and in a variety of neurodegenerative diseases, such as Alzheimer’s disease, Parkinson’s disease, and multiple sclerosis (Guo et al. 2021; Sarkar and Biswas 2021). These findings further suggest the possibility that A1/A2 astrocytes are important for disease onset and progression. In our current study, we also observed astrocyte polarization in the sensory-motor cortex in mice after SAH. The A1 astrocyte marker S100B was significantly elevated in mouse brain tissues after SAH, while S100A10 was decreased, demonstrating the polarization of astrocytes toward the neurotoxic phenotype after SAH. After injection with taurine, the expression of S100A10 was significantly increased, while the expression of S100B was decreased, demonstrating that taurine administration promoted the polarization of astrocytes toward the neuroprotective phenotype. Therefore, altering the polarization of astrocytes may be a mechanism by which taurine exerts neuroprotective effects.

## Conclusion

In the present study, we first prove that the taurine transporter is expressed most strongly on the membranes of neurons. After SAH, the expression of TauT was higher than that in normal neurons. Taurine supplementation obviously improved the sensorimotor function of mice after SAH. In addition, taurine ameliorated the cortical neuron damage caused by SAH. We also prove that taurine administration reduced SAH-induced neuronal apoptosis. Furthermore, we found that the proportion of A2 astrocytes was enhanced by taurine administration.

## Data Availability

All data included in this study are available upon request by contact with the corresponding author.
